# Rapid Adaptation to the Timbre of Natural Sounds

**DOI:** 10.1038/s41598-018-32018-9

**Published:** 2018-09-14

**Authors:** Elise A. Piazza, Frédéric E. Theunissen, David Wessel, David Whitney

**Affiliations:** 1 Princeton Neuroscience Institute, Princeton University, Princeton, NJ 08544 USA; 2 Helen Wills Neuroscience Institute, University of California, Berkeley, Berkeley, CA 94720 USA; 3 Vision Science Graduate Group, University of California, Berkeley, Berkeley, CA 94720 USA; 4 Department of Psychology, University of California, Berkeley, Berkeley, CA 94720 USA; 5 Department of Music, University of California, Berkeley, Berkeley, CA 94720 USA; 6 Center for New Music and Audio Technologies, University of California, Berkeley, Berkeley, CA 94720 USA

**Keywords:** Auditory system, Sensory processing, Human behaviour

## Abstract

Timbre, the unique quality of a sound that points to its source, allows us to quickly identify a loved one’s voice in a crowd and distinguish a buzzy, bright trumpet from a warm cello. Despite its importance for perceiving the richness of auditory objects, timbre is a relatively poorly understood feature of sounds. Here we demonstrate for the first time that listeners adapt to the timbre of a wide variety of natural sounds. For each of several sound classes, participants were repeatedly exposed to two sounds (e.g., clarinet and oboe, male and female voice) that formed the endpoints of a morphed continuum. Adaptation to timbre resulted in consistent perceptual aftereffects, such that hearing sound A significantly altered perception of a neutral morph between A and B, making it sound more like B. Furthermore, these aftereffects were robust to moderate pitch changes, suggesting that adaptation to timbral features used for object identification drives these effects, analogous to face adaptation in vision.

## Introduction

Simple features of sounds, such as frequency and intensity, are well studied and provide important cues about the world. But to experience the rich diversity of sounds in natural scenes, we rely on more than these low-level features. Timbre is a complex perceptual property that is essential for our discrimination, recognition, and enjoyment of sounds. It is often broadly defined as the unique quality or color of a sound that creates a strong link to the identity and state of its source and cannot be attributed simply to pitch, intensity, duration, or location^1^. For instance, Tom Waits’ gravelly growl is clearly distinguishable from Barry White’s velvety voice, even when they are singing the same pitch. Individuals modulate their vocal fingerprints to communicate with infants^2^ or convey different moods, and listeners are sensitive to even mild affective changes signaled by these timbral shifts^3^. The bright buzz of a muted trumpet, the harshness of screeching tires, the nasality of Fran Drescher’s laugh, the pleading intensity of an opera singer’s vibrato, and the formants in speech are all timbral properties that allow us to distinguish voices and objects, understand human language, and enjoy music. Timbre lends vividness not only to our perceptual experiences but also to our memories of sounds. For instance, people can identify detailed information about musical emotion and genre from very brief (300-ms) clips of popular music; this ability suggests that timbre contributes critically to schemata of musical genre, since these short samples contain very limited information about melodic, harmonic, bass, and rhythmic features^4,5^. Although the importance of timbre in our everyday auditory experiences is well documented, perceptual adaptation to this cue has not been thoroughly explored. A few studies have separately investigated adaptation to individual dimensions of voice perception^6,7^ or to a single pair of musical instruments^8^. However, it is unknown whether timbre adaptation generalizes to a broader range of natural sounds, musical instruments, and human voices, whether it is at all robust to changes in pitch, or whether it is driven by a single timbre feature, such as spectral centroid. If timbre is selectively adaptable, such that object- or source-specific negative aftereffects occur following exposure to the timbre of a particular auditory object, this suggests it is encoded as an explicit, holistic property that points to the identity of natural sounds. Furthermore, because timbre helps us continuously track objects (e.g., we can follow an oboe solo within a sea of woodwind instruments even as the soloist constantly changes pitch), this adaptation should be to some degree robust to changes in other auditory features such as pitch.

Adaptation, the reduction in a neural or behavioral response following prolonged exposure to a stimulus, is a critical and widespread mechanism in perception that facilitates interpreting stimuli by recalibrating the brain’s response to the current environment. Adaptation is present at multiple levels of the auditory pathway, from the inferior colliculus^9^ to the auditory cortex^10^ (see^11^ for a review) and operates at multiple time scales^12^. It is known to occur for multiple simple features of sounds, including frequency, level^10^, and duration^13^. In addition, auditory midbrain neurons adapt to statistics of the environment, rapidly adjusting their responses to probabilistic changes in intensity to improve population coding of the most likely levels^14^. Similarly, the overall spectral range of a distribution of sinusoidal tones impacts the spread of adaptation in auditory cortex^15^. However, although adaptation to simple features and statistical properties of artificial sound distributions is well studied, it is unknown whether humans perceptually adapt to the more complex combination of spectro-temporal features—the timbre—of natural sounds.

In the present study, we measured adaptation to a variety of sounds. Specifically, we quantified perceptual aftereffects by comparing listeners’ judgments of probes (intermediate morph steps between two distinct, complex adapter sounds) across three conditions: baseline (no adaptation), repeated exposure (adaptation) to one of the sounds in an adapter pair, and repeated exposure to the other sound in the pair. The class of adapter sounds varied across experiments, including musical instrument tones controlled for all non-timbre features, and natural sounds, varying from human voices to bird chirps to environmental textures (e.g., rain and wind). We found robust perceptual aftereffects, measured as shifts in the perceptual interpretation of morphs, resulting from adaptation to a variety of complex instrument and natural sounds.

## Results

### Adaptation to the timbre of natural sounds results in robust perceptual aftereffects

**Figure 1:**
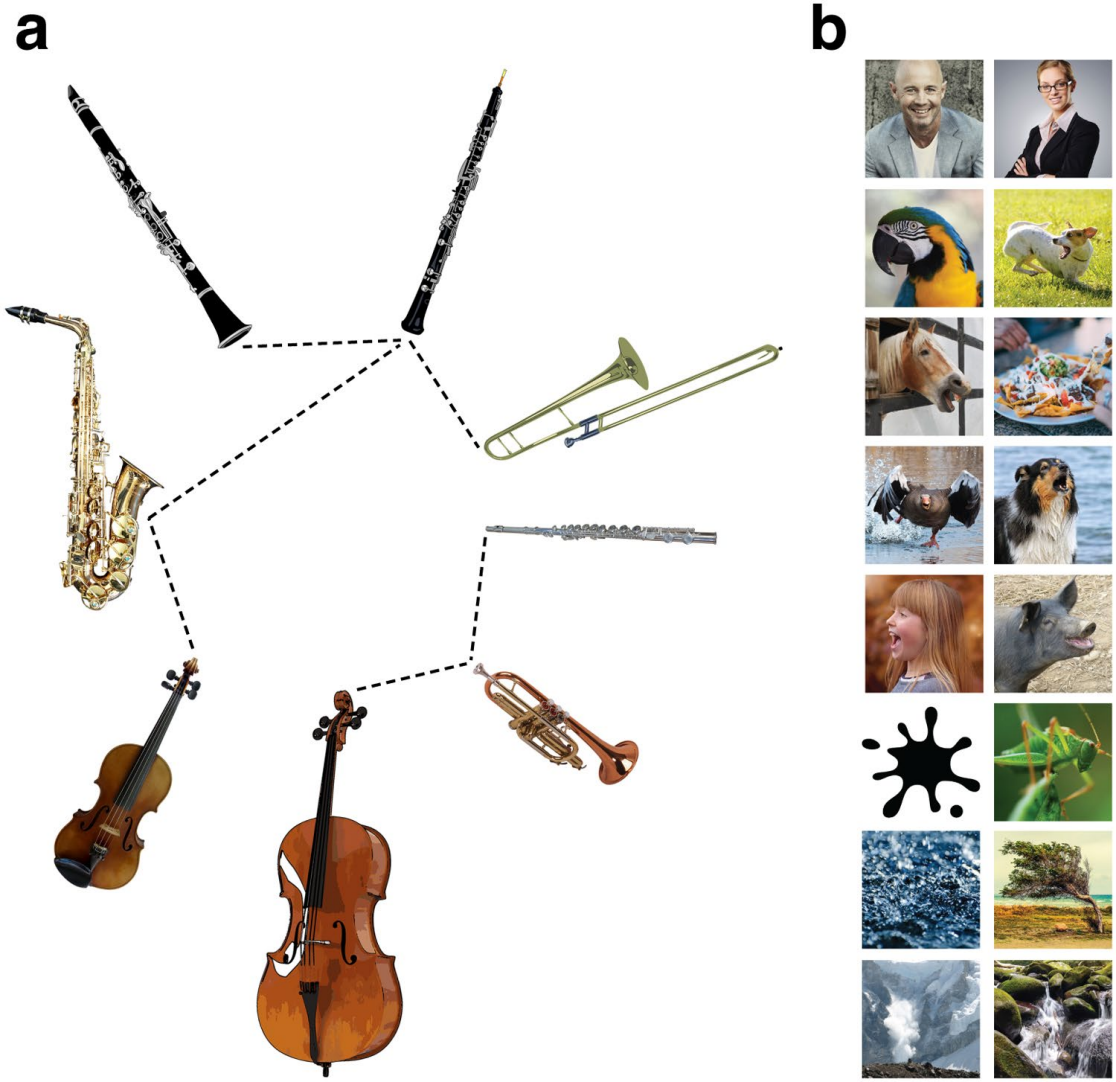
(**a**) Full set of instruments used as adapter sounds in Experiment 1. All instrument sounds came from the McGill University Master Samples collection^52^ and were equated for duration, level, and pitch. Dotted lines indicate morphed adapter pairs. (**b**) Images depicting the full set of natural sounds used as adapters in Experiment 2. Each row is a morphed pair.

**Figure 2:**
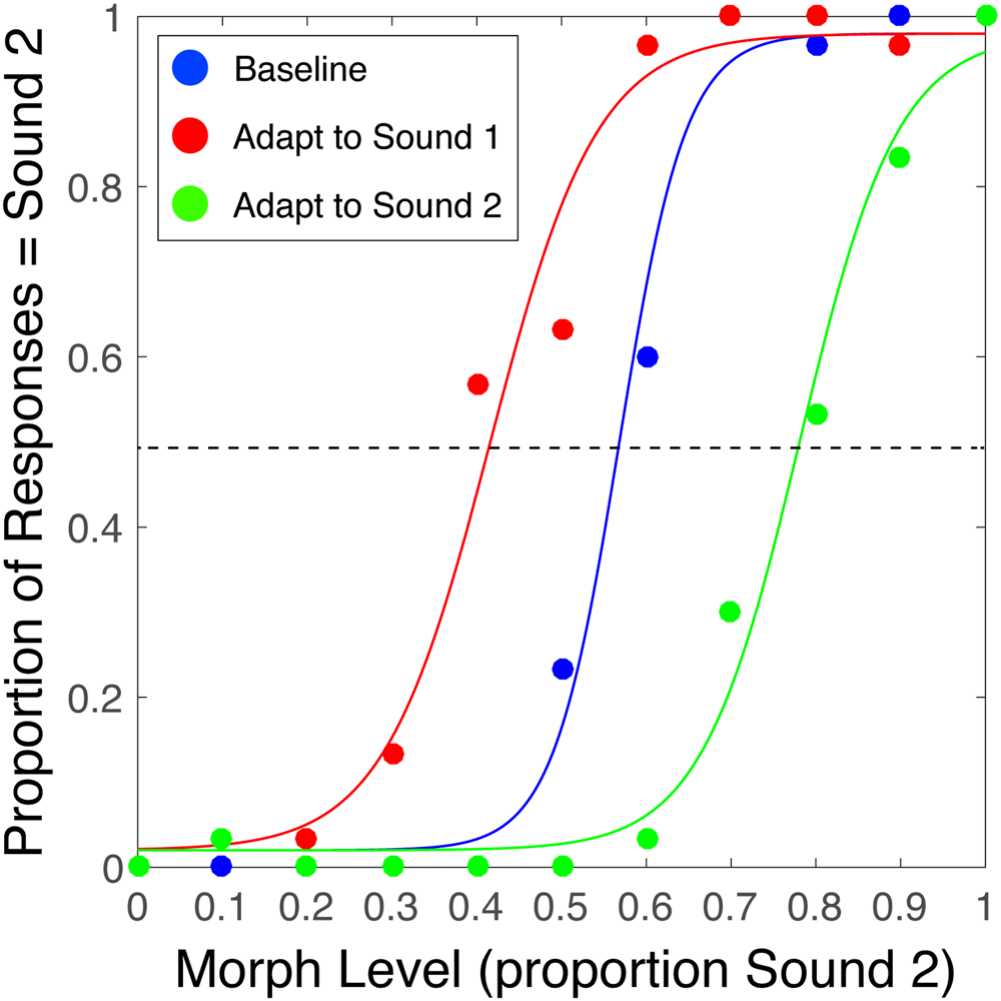
Psychometric functions for a single representative participant in Experiment 1. In each trial of every experiment, participants heard one of the morph levels between the two sounds in a pair and identified which sound they heard (sound 1 or sound 2). A morph level of 0 comprises only sound 1, a morph level of 1.0 comprises only sound 2, and a morph level of 0.5 is a mixture midway between the two (see Methods). In each of the three conditions (“baseline”, “adapt to sound 1”, “adapt to sound 2”), we fit a logistic function to the data. See Supplementary Information for analysis of goodness-of-fit across participants. The PSE of each curve is the x-axis value at which the curve crosses the dotted line.

We investigated the effects of adaptation (through repeated exposure to two distinct adapter sounds) on subsequent auditory perception. We generated sets of sound morphs between pairs of adapter sounds (for example, a clarinet and an oboe; see Methods and Fig. 1) and tested listeners’ perception of those morphs in a “baseline” condition (no adaptation) and two adaptation conditions (“adapt to sound 1”, “adapt to sound 2”). In each adaptation trial, the participant heard one of the adapter sounds (e.g., clarinet) five times and then had to categorize a morph (e.g., a 50% mixture between a clarinet and oboe; see Methods and Fig. 2) as one of the two sounds. Each adapter (e.g., an Eb4 played on an oboe, a chirp, a spoken syllable) was an auditory event whose timbre pointed to a source category or identity (an oboe, a bird, a specific person). Each baseline trial contained the same categorization task but the participant heard no adapter sound before making a judgment.

**Figure 3:**
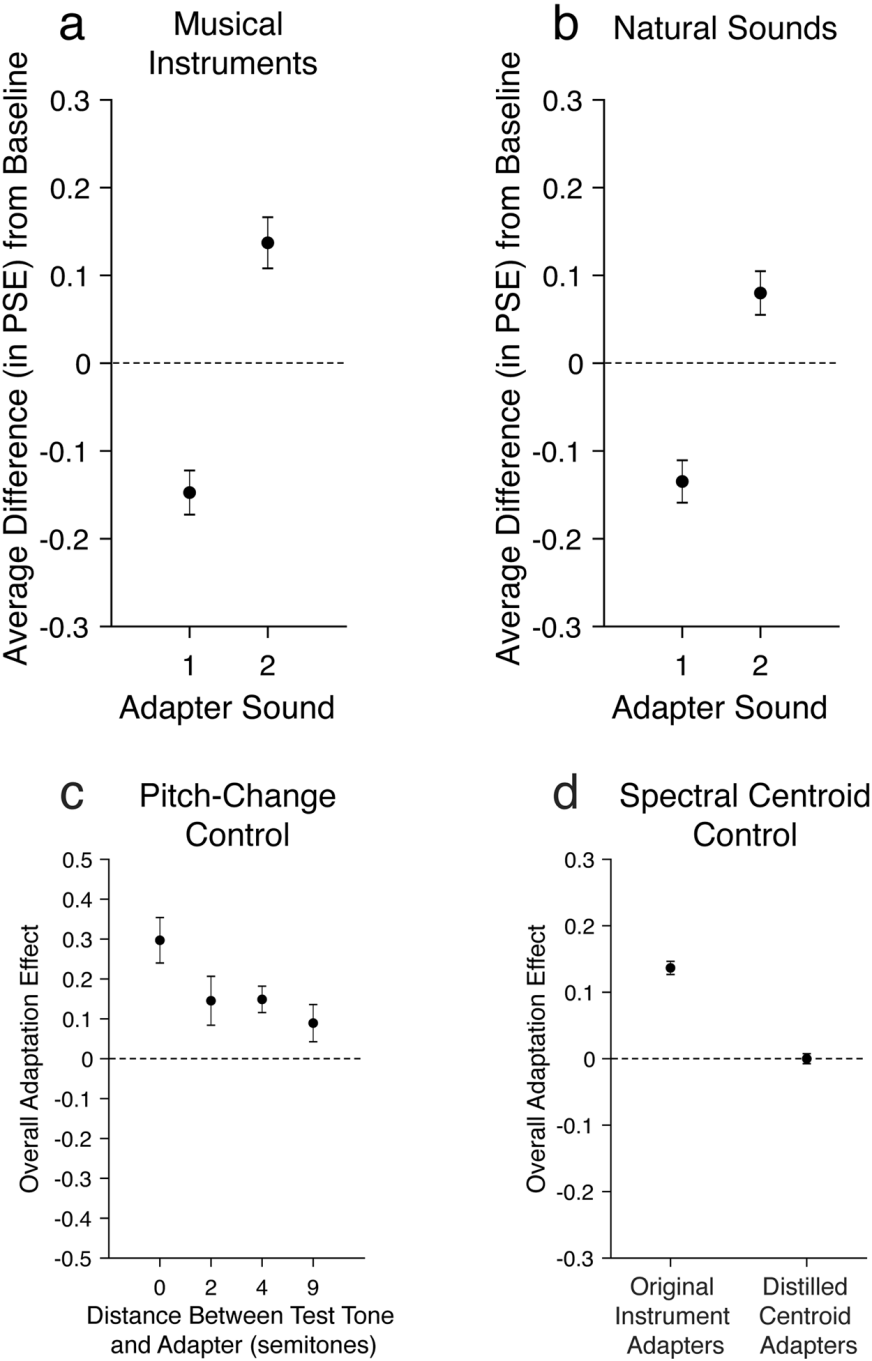
Mean adaptation effects in all experiments. For each subject, we computed the difference in PSE between the “adapt to sound 1” and “baseline” conditions (a measure of the perceptual aftereffect of adapting to sound 1; see Fig. 2) and between the “adapt to sound 2” and “baseline” conditions (**a** measure of the perceptual aftereffect of adapting to sound 2). The average aftereffect across subjects is shown here for each adaptation condition in (**a,b**), while (**c,d**) show the average overall adaptation effect (collapsed across adapters 1 and 2). (**a**) Experiment 1 (N = 16). (**b**) Experiment 2 (N = 30). (**c**) Mean overall adaptation effects in Experiment 3 for four different pitch changes of the test morph, relative to the adapter sounds: 0 (same pitch; Eb4, or 311 Hz), 2 (F4, or 349 Hz), 4 (G4, or 392 Hz), and 9 (C5, or 523 Hz). Higher scores indicate stronger aftereffects. N = 12. (**d**) Mean overall adaptation effects in Experiment 4a and 4b. Each participant completed one condition that was identical to Experiment 1 (the clarinet-oboe pair; left panel) and another condition in which the adapters were harmonic stacks with spectral centroids equivalent to the original instrument adapter sounds from Experiment 1 (right panel). N = 12. All error bars are SEM.

Each subject adapted to only one of the relevant adapter pairs in their assigned experiment (i.e., one of the six possible instrument pairs in Experiment 1 (Fig. 1a), one of the eight possible natural sound pairs in Experiment 2 (Fig. 1b)). Multiple subjects adapted to each pair (Supplementary Fig. S3). In Experiments 1 and 2, we combined data from all sounds (i.e. data from all adapter pairs; Fig. 3a,b) since individual stimulus pairs did not belong to particular groups, were not designed to test specific hypotheses about particular sounds, and yielded highly consistent results (Supplementary Fig. S3).

For each subject, we first separately measured the perceptual aftereffect in each adaptation condition by subtracting the point of subjective equality (PSE) of the psychometric function (see Methods and Fig. 2) of the “baseline” condition from the PSEs in the “adapt to sound 1” and “adapt to sound 2” conditions. Group-averaged difference values are shown in Fig. 3a,b. Next, we quantified the strength of the overall adaptation effect for each subject by collapsing these two difference values into a single value (specifically, by flipping the sign of one of the difference values and averaging it with the other). The overall adaptation effect corresponds to the degree to which adaptation altered participants’ perceptual interpretation of the morphs in the expected direction (e.g., adapting to sound 1 makes a neutral 50% morph sound more like sound 2 and vice versa); higher (more positive) values indicate stronger adaptation. Individual subject values are shown in Supplementary Fig. S3. We computed a group overall adaptation effect by averaging the overall adaptation effect values across subjects. In Experiments 1 and 2, respectively, this group overall adaptation was significantly greater than zero for combined data from all instrument pairs (two-tailed one-sample t-test; *t*(15) = 6.87, *p* < 0.0001, Cohen’s *d* = 1.72) and for combined data from all natural sound pairs (two-tailed one-sample t-test; *t*(29) = 6.33, *p* < 0.0001, Cohen’s *d* = 1.16).

### Timbre adaptation transfers across moderate changes in pitch

At a crowded party, it is quite easy to track a friend’s unique voice identity, even though her pitch is constantly changing and often overlaps with other speakers’ pitches. If the aftereffects we found reflect adaptation of neural populations responsive to the timbral qualities that point to an auditory object’s identity, they should be to some degree robust to variations in the object’s acoustic features, such as pitch changes that ubiquitously occur in the speech produced by an individual speaker. In Experiment 3, we investigated this possibility by varying the fundamental frequency of the probe sound across trials (Fig. 3c). Specifically, we used clarinet and oboe samples that were naturally recorded on different pitches (either 0, +2, +4, or +9 semitones from the original Experiment 1 adapters), equated their duration and intensity, and generated four new morph sets between these sounds (see Methods). The experiment was identical to Experiment 1 except that in each of four blocks, the test sounds were sampled from one of these four sets. The adapter sounds were always clarinet and oboe sounds played on Eb4 (the same pitch as the 0-semitone shift test sounds). Every participant completed all four blocks, and the order of these blocks was counterbalanced across participants. We found significant overall adaptation effects for pitch changes (relative to the adapters) of 0 semitones (two-tailed paired t-test, *t*(11) = 5.21, *p* < 0.001), 2 semitones (*t*(11) = 2.37, *p* < 0.05), and 4 semitones (*t*(11) = 4.49, *p* < 0.001) and a marginally significant effect for a pitch change of 9 semitones (*t*(11) = 1.91, *p* = 0.08). Because timbre adaptation is robust to changes in the local features caused by changing pitch by up to at least 4 semitones, we can conclude that it cannot be explained by simple shifts in the gain of local information (e.g. here in specific individual frequency bands corresponding to the fundamental and its overtones), because that information changes drastically with even the smallest pitch changes, while the overall timbral structure remains largely intact (coarse spectral envelope, temporal envelope, etc.). It should be noted that participants’ discrimination of the two instrument categories (specifically, the slope of the psychometric function in the baseline condition) did not differ significantly across the four pitch conditions (one-way ANOVA, *F*(3,11) = 0.84, *p* = 0.48), indicating that the effect of pitch changes on the timbre aftereffect was not due to altered discriminability of the two instrument identities.

### Adaptation to an isolated timbre feature does not bias perception of natural sounds

If perceiving the identity of an object through timbre information requires integrating features into a holistic percept, then adapting to one single dimension of timbre should not result in perceptual aftereffects on natural sound perception like the effects we report in Experiments 1 and 2. In Experiment 4, we generated artificial sounds based on the spectral centroid of the clarinet and oboe tones from Experiment 1. We selected spectral centroid because it has been used as a representative timbre feature^16^ and shown to be one of the primary features driving timbre perception^17–21^. If adaptation to this single timbre feature generates aftereffects for natural sounds, the perceptual aftereffects we report in Experiments 1–3 may be due to adaptation to individual, independent features or dimensions of timbre. If we instead find that listeners do not adapt to this isolated dimension, this would suggest that adaptation requires integrating multiple timbre features, likely both spectral and temporal, into a holistic percept of a sound source. A single group of participants completed two conditions (Fig. 3d): in one (Experiment 4a), they adapted to the original (natural) clarinet and oboe sounds (left panel), and in the other (Experiment 4b), they adapted to the new artificial centroid-based sounds (right panel). This second condition was designed to test whether an isolated feature of timbre could shift perception of the natural timbres from which that dimension was extracted. In both conditions, they were tested on morphs between the original natural sounds (the clarinet and oboe from Experiment 1).

We found a significant overall adaptation effect in the first condition, which was a direct replication of Experiment 1 (“original instrument” adapters; two-tailed one-sample t-test; *t*(11) = 13.8, *p* < 0.0001, Cohen’s *d* = 3.99; Fig. 3d, left panel), but not in the second condition (“distilled centroid” adapters; *t*(11) = 0.00, *p* = 0.99, Cohen’s *d* = 0.00; Fig. 3d, right panel). A paired (within-subject) comparison between the two conditions confirmed that the strength of the overall adaptation effect was significantly larger in the “original instrument” than the “distilled centroid” condition (two-tailed paired samples t-test; *t*(11) = 11.9, *p* < 0.0001, Cohen’s *d* = 3.44).

To eliminate the possibility that the lack of adaptation to the distilled sounds in Experiment 4b could be due to a general failure of subjects to adapt to these stimuli at all, in any context (possibly due to perceptual indiscriminability), we also confirmed that this isolated centroid dimension was adaptable on its own in Supplementary Experiment 1 (Supplementary Fig. S4).

### Response bias is unlikely to drive our results

One might wonder if response bias were responsible for the measured aftereffects. This is unlikely. First, if response bias (and not perceptual aftereffects) were driving our findings, we would expect that repeated exposure to an instrument category would shift responses to test morphs of all pitches in Experiment 3 somewhat equally—that there would be no tuning for the test stimulus pitch. This was not the case: the aftereffect declined from 0-semitone to 9-semitone shifts (Fig. 3c; one-way ANOVA, *F*(3,11) = 7.73, *p* < 0.001), suggesting that a systematic response bias is not likely responsible for the results. Moreover, the effects we report are quite large—on average, around 2 JNDs in each direction (3–4 JNDs total between the two adaptation conditions)—indicating that listeners can simply experience the effect; like the motion-, pitch- or other large aftereffects, this timbre aftereffect is a noticeable illusion (see Supplementary Audio S13 for a demonstration). Previous literature has demonstrated that even when participants are asked to intentionally bias their responses (absent of perceptual adaptation), the PSE can only be shifted by relatively small amounts (i.e., a small fraction of the spread of the psychometric function^22^;) without changing slope, and we report larger shifts but find no evidence of changes in slope between “baseline” and either “adapt to sound 1” trials (Experiment 1, *t*(15) = −1.18, *p* = 0.25; Experiment 2, *t*(29) = −0.99, *p* = 0.33) or “adapt to sound 2” trials (Experiment 1, *t*(15) = −0.82, *p* = 0.43; Experiment 2, *t*(29) = −0.92, *p* = 0.36).

### Time course analysis

**Figure 4:**
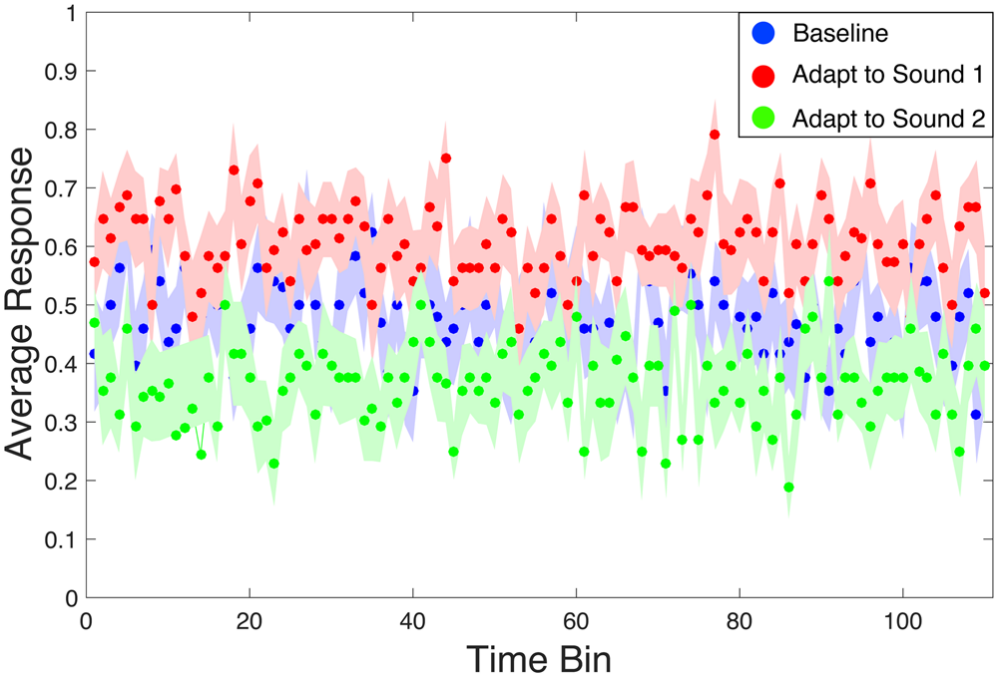
Time course of perceptual aftereffects in Experiment 1. We divided each block (e.g., “baseline”) into bins of three trials and averaged the responses (sound 1 or sound 2) in each bin. A value of “0” represents a response to sound 1 and a value of “1” represents a response to sound 2. Each point represents the mean of these average responses across subjects. N = 16. Shaded error bars are SEM.

To verify that the aftereffects we found were present immediately (within a single trial) and not driven by long-term adaptation that developed over the course of an experimental session, we analyzed the strength of adaptation across the entire block for each subject in Experiment 1. Specifically, we divided each full block (e.g., “baseline”) into 110 time bins, each containing three trials, and averaged the responses in each bin. An average response close to 1 indicates perceiving sound 1 more often than sound 2, and an average response close to 2 indicates perceiving sound 2 more often. Figure 4, which depicts the development of this average response across time (averaged across subjects), demonstrates that the aftereffect is robust from the very first trial of the experiment, immediately following initial adaptation. If there were no adaptation, the responses corresponding to the adaptation conditions (green and red data in Fig. 4) would overlap the baseline responses (blue data).

## Discussion

We found that listeners quickly adapt to the timbre of a variety of natural sounds. Furthermore, the perceptual aftereffects we report were robust to moderate pitch changes (up to at least 4 semitones) between the adapter and test stimulus, indicating that adaptation can survive much of the pitch variability present in natural sounds (voices, animal calls, etc.). These findings represent the first demonstration of robust and widespread aftereffects from adaptation to auditory timbre. These significant shifts in the perceptual interpretation of sounds based on recent context represent a noticeable illusion that can be experienced within a single trial (see Supplementary Audio S13 for a demonstration).

Timbre conveys critical information about the identity of a sound and allows us to distinguish musical instruments, animals, the gender, age or emotional expression of voices, the identity of vowels in speech, and many other biologically relevant sounds. For instance, radio voice artists are classified by the unique color of their voice, based on dimensions such as breathiness, roughness, or harshness^23^. The rapid adaptation we report likely enables us to automatically adjust our perception to the spectro-temporal profile of the current auditory environment in order to improve our sensitivity to novel or unusual stimuli. For instance, by adapting to a friend’s voice during a conversation, we become better equipped to notice when a new speaker with different timbral characteristics enters the room. Composers likely exploit this adaptation effect, whether consciously or not, since it helps to amplify musical contrast. The results of Experiment 3 suggest that this adaptation can, to some degree, survive the pitch shifts that occur in sounds produced by a given auditory source and, therefore, that the underlying mechanisms could also play an important role in identifying and maintaining representations of sound sources. Timbre adaptation may also afford auditory constancy across a lifetime of changes to our perceptual systems (e.g., due to hearing loss) or our own vocal quality.

Here, we have adopted the canonical conceptualization of timbre in the field^1,24^, as a characteristic of sounds—aside from pitch, intensity, duration, and location—that contributes to the identification of sound sources and to the grouping of auditory objects. This said, timbre need not be equated to sound source identity: a particular sound source (e.g., a human voice, a violin) can emit multiple timbres (different vowels, bowed vs. plucked sounds). Playing an instrument in an unusual or extreme manner, thereby producing unorthodox timbres (e.g., prepared piano, muted brass, woodwind squeaks, flutter tongue) can make that instrument difficult to recognize, similar to how an extreme facial expression^25^ or makeup^26^ can hinder face recognition. Pitch can also contribute to the identity of a sound, but characterizations of the relationship between pitch and timbre are somewhat murky. Some studies describe interactions and interference between the two features^27,28^, others report a very limited relationship for small pitch changes^29,30^, and although the cortical regions most responsive to variations in the two features largely overlap, they nonetheless drive separable patterns of activation at the single-subject level^31^. It does seem clear that large pitch differences (beyond one octave) can make it difficult to determine whether two sounds came from the same or different instruments^29,30^. Our observation in Experiment 3 of a gradually reduced aftereffect for increasing pitch shifts of the test stimuli strengthens the view that timbre provides a consistent pointer to a particular auditory object until pitch shifts significantly.

Natural sound perception depends on holistic processing in similar ways to the perception of complex visual objects, such as faces. Just as a loved one’s face is instantly recognizable across a variety of sizes, orientations, or contrasts, the same person’s voice retains its unique identity or Gestalt across a range of pitches and durations and a wide variety of utterances^32^. Our finding of pitch tuning (i.e., the decline in aftereffect strength for increasing pitch shifts) is somewhat analogous to demonstrations that high-level adaptation to (and recognition of) the holistic identity of faces^33,34^ are tuned to other image features, such as size^35^, retinal position^36–38^, and orientation^39,40^. Although pitch shifts larger than 9 semitones are fairly common in everyday melodic perception, they are typically interspersed between much smaller shifts^41^, which may facilitate persistent adaptation. Relatedly, naturalistic contexts provide many additional cues (e.g., continuity in amplitude, location, and in the visual stimulus), that likely help to bridge object identity across large pitch shifts.

Human voices are biologically relevant natural stimuli that are detected quite quickly based on their rich timbre information^42^. Several previous studies have investigated adaptation to the gender^6,43^, emotional tone^43,44^, identity^45^, and age^46^ of human voices by measuring behavioral aftereffects of adapting to one of two extremes on a particular perceptual dimension (e.g., gender). Many of these studies did not match the pitch of the adapters and thus may be due partly to adaptation to F0, rather than timbre alone. Here, we matched the pitch, duration, and intensity of the instrument sounds (Experiment 1) and the human voice sounds (Experiment 2) to eliminate the possibility that the aftereffects we found were due to these features. One study^7^ did investigate adaptation to vocal gender while controlling for F0, and there is additional evidence for effects of spectral context on speech perception (e.g., vowel identification^47^;). However, our study investigates the more general phenomenon of timbre adaptation across a broad range of sound categories, similar to^33^, which reported a general principle for face adaptation across many categories after individual variants of the effect had been previously demonstrated. More importantly, in contrast to previous studies, we found that the perceptual aftereffect following timbre adaptation generalized across moderate changes in pitch, demonstrating that this phenomenon can operate in naturalistic environments.

The timbre adaptation we describe relies on the integration of multiple acoustic features, including frequency, intensity, temporal modulation, and spectral timbre features, into a coherent object, as demonstrated by Experiment 4, which showed that exposure to a single isolated dimension of timbre (spectral centroid) does not alter perception of the natural sound from which it was extracted. Spectral centroid has been consistently found to be one of the most important drivers of timbre perception^17,19^ and has been used as a representative timbre feature^16^. Because exposure to spectral centroid alone failed to shift listeners’ perception of natural sounds at all (Fig. 3d, right panel), it is therefore highly unlikely that exposure to a different single timbre dimension (e.g., attack time) would have elicited aftereffects large enough to fully account for the results we report for natural sound adaptation (Cohen’s *d* = 3.99; Fig. 3d, left panel). However, in the interest of better understanding how timbre is coded, future work might more thoroughly test whether any combination of timbre dimensions can produce the aftereffects we report for complex natural sound classes, or whether these effects result from adaptation to an emergent holistic object property and cannot be explained by a straightforward sum of features. Of course, we do also adapt to simpler auditory features in isolation, such as frequency, level^10^ and spectral centroid (see Supplementary Experiment 1). Two additional hypotheses to explore in future work are whether adaptation to natural sound classes yields stronger aftereffects than adaptation to simple features, and whether the more complex mental representations driving the adaptation of natural sound classes are reflected in higher-order brain areas than the representations of simpler features.

In conclusion, we find that human listeners perceptually adapt to the timbre, or unique identity, of a wide range of natural sounds. This adaptation could help achieve timbre invariance, which would enhance our sensitivity to novel or rare auditory objects, such as a new speaker’s voice in a conversation. Importantly, this adaptation survives moderate shifts in pitch, a feature that naturally varies (largely independently of timbre) in the environment. Timbre information—the raspiness or purity of someone’s voice, the smoky, Harmon-muted trumpet of Miles Davis, the percussive crack of a snare drum, and the rough screech of a barn owl—drives the richness of auditory perception and memory. Adaptation to this timbre information could help the auditory system recalibrate itself to the predominant signals in the environment, and in so doing serve as a critical and flexible cue to the object identity of sounds.

## Methods

### Participants

Seventy participants (ages 18–39) completed this study. Sixteen listeners (nine female) completed Experiment 1, thirty (sixteen female) completed Experiment 2, twelve (ten female) completed Experiment 3, and twelve (ten female) completed Experiment 4. All participants were naïve (i.e., they were given no information about the hypotheses, methods, or any other aspects of the study before beginning the experiment). According to self-report, all participants had normal hearing, and their degree of musical training varied widely (from no training to more than ten years of training on multiple instruments). All participants provided informed consent, all experimental protocols were approved by the Committees for the Protection of Human Subjects at the University of California, Berkeley and Princeton University, and the study was performed in accordance with the approved guidelines. We originally collected data from 82 participants but excluded twelve subject data sets from analysis (ten due to at-chance discrimination performance in the “baseline” condition and two due to key mapping errors). Our sample sizes were based on previous studies of auditory aftereffects^6,42,43^.

### Stimulus presentation

Sounds were presented in a sound-attenuated booth, over Sennheiser HD-280 Pro headphones at a fixed level (approximately 70 dB SPL) set at the beginning of each experiment. Participants were asked whether this was comfortable during the Instructions phase, and only a few participants chose to lower the volume to approximately 65 dB SPL. Stimuli were presented, and keyboard responses recorded, using the Psychophysics Toolbox^48^ in Matlab 7.11.0 (R2010b) on a computer running Mac OSX.

### Adapter selection and pre-processing

In each experiment, a different class of adapting stimuli was used. In Experiment 1, each participant adapted to a pair of musical instrument sounds (Fig. 1a), drawn from a set that spanned the space of instrument families and the two most significant dimensions of timbre discrimination found by^49^ (see Supplementary Fig. S1). All instrument sounds were equated in fundamental frequency (311 Hz), duration (1 second) and sound level. In Experiment 2, each participant adapted to a pair of natural sounds (Fig. 1b). See Supplementary Information, including Supplementary Audios S1–S15, for additional stimulus details and sound examples.

In Experiment 3, the pitch of the adapter sounds was always Eb4 (as in Experiment 1), but the pitch of the probe sound varied across blocks (see Experimental Procedure and Supplementary Audio S16). Each participant adapted to a clarinet-oboe pair from the University of Iowa musical instrument samples collection^50^. To create the sets of probe morphs, we used clarinet and oboe sounds from the same collection that were naturally recorded on either Eb4, F4, G4, or C5 (which varied by 0, +2, +4, or +9 semitones, respectively, from the adapter stimuli).

In Experiment 4, each participant completed two blocks (4a and 4b) in counterbalanced order (Fig. 3d). In one block (Fig. 3d, left panel), the adapters were the clarinet and oboe sounds from Experiment 1. In the other block (Fig. 3d, right panel), the adapters were two synthetic, harmonic sounds generated in Matlab from the spectral centroid (the weighted average frequency of a signal) and pitch of these two instrument sounds (F0 = 311 Hz, 20 harmonics, centroid of 630 Hz for clarinet and 890 Hz for oboe, with each harmonic weighted by its position within a Gaussian distribution centered on the spectral centroid). The test sounds, which were identical across the two blocks, were morphs between the original clarinet and oboe (i.e., the same test sounds used in Experiment 1). See Supplementary Information for detailed morphing procedures.

### Experimental procedure

In every block of every experiment, the participant’s task was to listen to a sound and categorize it (e.g., as a clarinet or oboe). Participants first completed a pre-test phase to familiarize them with the two possible categories: they were given the names of the sounds (e.g., “clarinet”, “oboe”, “dog”, “goose”), accompanied by the sounds themselves and photos of the corresponding sound sources. At the end of the pre-test phase in each experiment, participants completed an identification task in which they were given four repetitions of each of the two categories (in random order) and had to classify each sound with a key press. Any participant who did not achieve 100% performance on this task by the second attempt was excluded from the experiment.

In the next phase of each experiment, participants’ baseline perceptual discrimination was measured for the sounds in the assigned morph set. In each trial of this baseline phase, participants heard a probe sound (a randomly selected morph) and had to classify it as one of the two sound categories (two-alternative forced choice design). The proportion of responses to one sound was calculated and a psychometric (logistic) function was fit to the data using Palamedes^51^ (see Fig. 2). Psychometric functions for individual subjects in Experiment 1 are shown in Supplementary Fig. S2. The point of subjective equality (PSE) corresponds to the 50% threshold, or the morph that the subject was equally likely to classify as either of the two sound categories. The subsequent adaptation phase was identical to the baseline phase except that at the beginning of each trial (directly preceding the probe), one of the two adapter sounds was repeated five times. This adaptation phase was blocked by adapter: each participant first completed a block of trials in which one of the sounds was the adapter, followed by a block in which the other sound was the adapter. The order of these blocks was counterbalanced. We used a blocked design to minimize interference between trials containing different adapters. In every block of Experiments 1 and 2, the test sounds were randomly chosen from the spectrum of morphs between the two sounds that also served as adapters in that block. In Experiments 1 and 2, we randomly assigned a pair of adapters to each participant (e.g., “clarinet” and “oboe” in Experiment 1). In Experiment 3 only, the pitch of the probe (test) tone varied across four blocks, between Eb4 (311 Hz), F4 (349 Hz), G4 (392 Hz), and C5 (523 Hz), with block order counterbalanced across participants.

In each baseline and adaptation block, participants completed thirty repetitions at each morph level. Because of its length (~2 hours), Experiment 3 had twenty repetitions at each morph level. Each experiment lasted about 1.5–2 hours, which some subjects chose to split across two days (specifically, thirteen subjects in Experiment 1, two in Experiment 2, and four in Experiment 4).

## Electronic supplementary material

Supplementary Information

Audio S1

Audio S2

Audio S3

Audio S4

Audio S5

Audio S6

Audio S7

Audio S8

Audio S9

Audio S10

Audio S11

Audio S12

Audio S13

Audio S14

Audio S15

Audio S16

## Data Availability

The datasets generated and analyzed during the current study are available from the corresponding author on reasonable request.
