# Publisher Correction: Rapid Adaptation to the Timbre of Natural Sounds

**DOI:** 10.1038/s41598-020-59861-z

**Published:** 2020-02-21

**Authors:** Elise A. Piazza, Frédéric E. Theunissen, David Wessel, David Whitney

**Affiliations:** 1 Princeton Neuroscience Institute, Princeton University, Princeton, NJ 08544 USA; 2 Helen Wills Neuroscience Institute, University of California, Berkeley, Berkeley, CA 94720 USA; 3 Vision Science Graduate Group, University of California, Berkeley, Berkeley, CA 94720 USA; 4 Department of Psychology, University of California, Berkeley, Berkeley, CA 94720 USA; 5 Department of Music, University of California, Berkeley, Berkeley, CA 94720 USA; 6 Center for New Music and Audio Technologies, University of California, Berkeley, Berkeley, CA 94720 USA

Correction to: *Scientific Reports* 10.1038/s41598-018-32018-9, published online 14 September 2018

Supplementary files containing Audio S1-S16 were omitted from the original version of this Article. This has been corrected in the HTML version of the Article; the PDF version was correct at the time of publication.

