# Supplementary material for: Rapid Adaptation to the Timbre of Natural Sounds
Source: Sci Rep. 2018 Sep 14;8:13826. doi: 10.1038/s41598-018-32018-9 (PMC6138731; doi:10.1038/s41598-018-32018-9)
Supplement: Supplementary file 1 — Supplementary Information [file 41598_2018_32018_MOESM1_ESM.docx]

**Supplementary Information**

Rapid Adaptation to the Timbre of Natural Sounds

Elise A. Piazza, Frédéric E. Theunissen, David Wessel, and David Whitney

Stimulus details

The musical instrument pairs used in Experiment 1 (see Figure 1A and Supplementary Fig. S1) were as follows: a clarinet and oboe, a violin (with vibrato) and tenor saxophone, a trumpet and flute, a trombone and oboe, a cello and trumpet, and a tenor saxophone and oboe. All instrument sounds were sustained tones at E-flat in octave 4 (above middle C) selected from the McGill University Master Samples collection [52] and further processed (equated in F0, duration, and sound level) by Elliott, Hamilton, & Theunissen (2013).

The natural sound pairs used in Experiment 2 were as follows: a male and a female speaker saying the syllable “da” (Supplementary Audio S4-S6), a parrot and a small dog, a horse and a crunching sound (Supplementary Audio S7-S9), a goose and a dog (Supplementary Audio S10-S12), a woman screaming and a pig squealing, two insect-related sounds, rain and wind, and an avalanche and a babbling brook (Figure 1B). The natural sounds were downloaded from a variety of online sources (freesound.org, freesfx.co.uk, soundbible.com, audiomicro.com, and soundjay.com) or recorded in the lab, and the two sounds in each pair were then normalized for duration and RMS sound level in Matlab. The spoken syllables were additionally equated in F0 (Ab; 204 Hz) in Audacity. Although sound duration was matched between the two sounds in a given adapter pair, duration varied across pairs.

Morphing procedures

In each experiment, for each adaptation axis (dotted lines in Figure 1A, rows in Figure 1B), we generated a range of sound morphs between the two adapters in the pair. In Experiment 1, we used eleven morphed sounds at equally spaced steps and in all other experiments, we used five morphed sounds to minimize the duration of the experimental session, since this set still provided sufficient precision to reliably fit psychometric functions to the data.

In Experiment 1, to morph the harmonic instruments, we used Loris, a software package that uses reassigned bandwidth-enhanced additive sound modeling to transform sounds [53]. To morph two harmonic source sounds, Loris first extracts the partials from each sound and then interpolates the time-varying frequencies, amplitudes, and bandwidths of corresponding partials in the sounds. In Experiment 2, to morph the natural sounds (many of which were non-harmonic), we used SoundHack, a similar software package that uses spectral mutation functions [54, 55] to “cross-fade” between two sounds. Specifically, it performs a series of Fast Fourier Transforms at multiple time points across the time course of each sound. At each time point (FFT frame), for each frequency band it combines the phase-amplitude pairs of the two sounds by weighting each pair by the relative contribution of that sound (e.g., for a 50% morph, the two sounds are weighted equally). This procedure generates a smooth morph even when the combined sounds contain substantial temporal variation, and it allowed us to include a wide variety of natural sounds in our stimulus set. To morph the spoken syllables, we used STRAIGHT [56, 57], a software package for morphing vocal sounds in Matlab. For each stimulus, STRAIGHT performs an instantaneous pitch-adaptive spectral smoothing to separate contributions from the glottal source and supralaryngeal filtering and decomposes the stimulus into a set of fundamental parameters that can be used for resynthesis, the values of which are interpolated between morphed sounds. We applied the different algorithms described above because they were optimally designed (and most frequently used) to morph the following types of sounds: harmonic sounds (Loris), voices (STRAIGHT), and non-harmonic sounds (SoundHack).

Goodness-of-fit of psychometric functions did not impact our results

For each adaptation condition (“adapt to sound 1”, “adapt to sound 2”), we measured the goodness-of-fit (specifically, deviance) of each participant’s psychometric function using the Palamedes Matlab toolbox [51, 58]. To determine the influence of quality of fit on our results, we performed a split-half comparison of the adaptation effects based on deviance. Specifically, we divided the dataset for each condition into “higher”- and “lower”- deviance groups and compared the strength of the perceptual aftereffect (i.e., the difference in the PSE from baseline) between these groups. We found no difference between the adaptation effects for the “high” and “low” deviance group in either the “adapt to sound 1” (Experiment 1: *t*(7) = -.97, *p* = .37; Experiment 2: *t*(14) = -.77, *p* = .45) or “adapt to sound 2” condition (Experiment 1: *t*(7) = -.81, *p* = .44; Experiment 2: *t*(14) = 1.01, *p* = .33). Thus, although there were slight differences in the quality of fits across participants, these differences did not impact our ability to capture robust shifts in the psychometric function due to adaptation.

The individual timbre dimension used in Experiment 4b (spectral centroid) is internally adaptable

To verify that the lack of adaptation to the distilled “spectral centroid” dimension in Experiment 4b was not simply due to participants’ general failure to adapt to these stimuli at all, in any context, we showed that this isolated centroid dimension was adaptable on its own in Supplementary Experiment 1. We exposed a separate group of twelve participants to the same distilled centroid adapters used in Experiment 4b and tested on morphs between them (instead of on morphs between the original clarinet and oboe, as in Experiment 4b). We generated the sound morphs between the distilled centroid adapters using SoundHack (see Morphing Procedures above). The experimental procedure was the same as in Experiment 1, except that the labels given for the two synthetic sound categories were “A” and “B”, arbitrarily assigned to the two endpoints of the dimension.

This experiment yielded statistically significant group overall adaptation effects (Supplementary Fig. S4; two-tailed one-sample t-test; *t*(11) = 10.70, *p* < .0001, Cohen’s *d* = 3.09). Thus, although Experiment 4b showed that naturalistic timbre adaptation cannot be explained by adaptation to a single core component of timbre (spectral centroid), that component on its own generates adaptation for its perception.


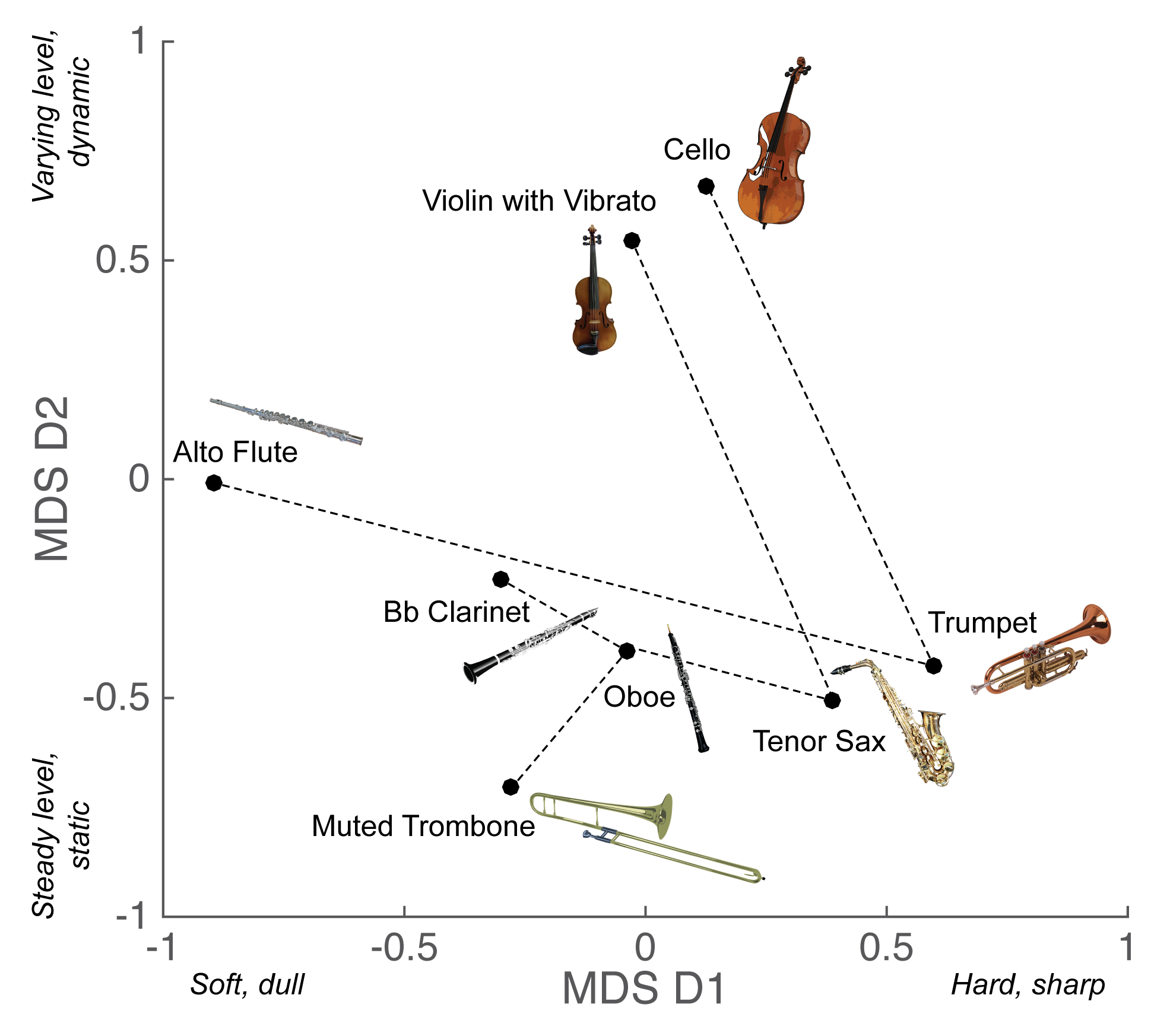


**Supplementary Figure S1**. Full set of instruments used as adapter sounds in Experiment 1. Axes correspond to the two most significant dimensions of timbre discrimination found by Elliott, Hamilton, & Theunissen (2013; see Figure 9A). All instrument sounds came from the McGill University Master Samples collection [52] and were equated for duration, level, and pitch. Dotted lines indicate morphed adapter pairs.


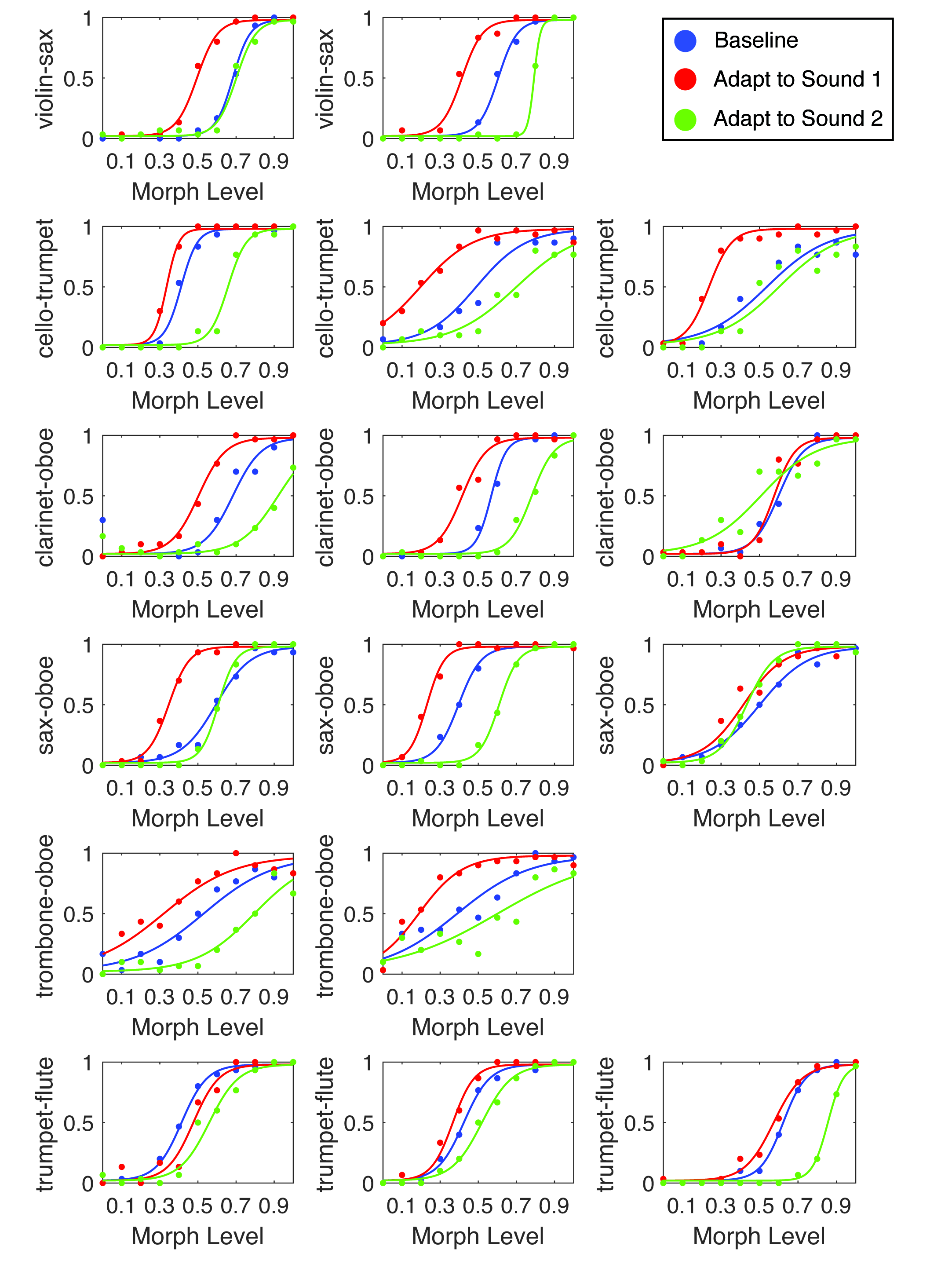


**Supplementary Figure S2**. Individual psychometric functions for all participants in Experiment 1. Each row corresponds to a morphed instrument pair.


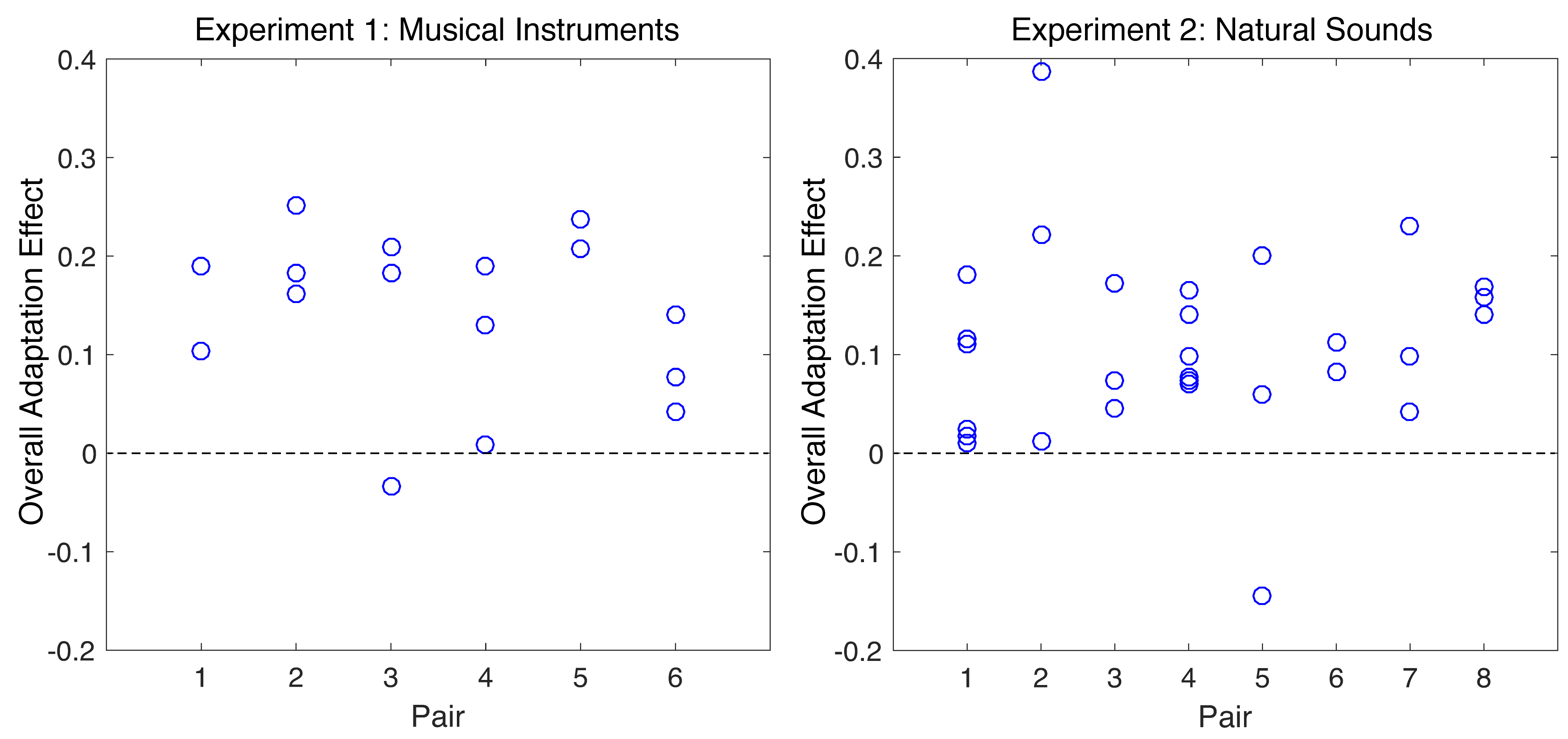


**Supplementary Figure S3**. Overall adaptation effects for all pairs in Experiments 1 and 2. Each blue circle corresponds to a single subject. Higher scores indicate stronger adaptation effects (collapsed across the “adapt to sound 1” and “adapt to sound 2” conditions; see Results). These effects were highly consistent and reliably above chance across pairs. Experiment 1 points derive from the individual psychometric functions shown in Supplementary Fig. S2.


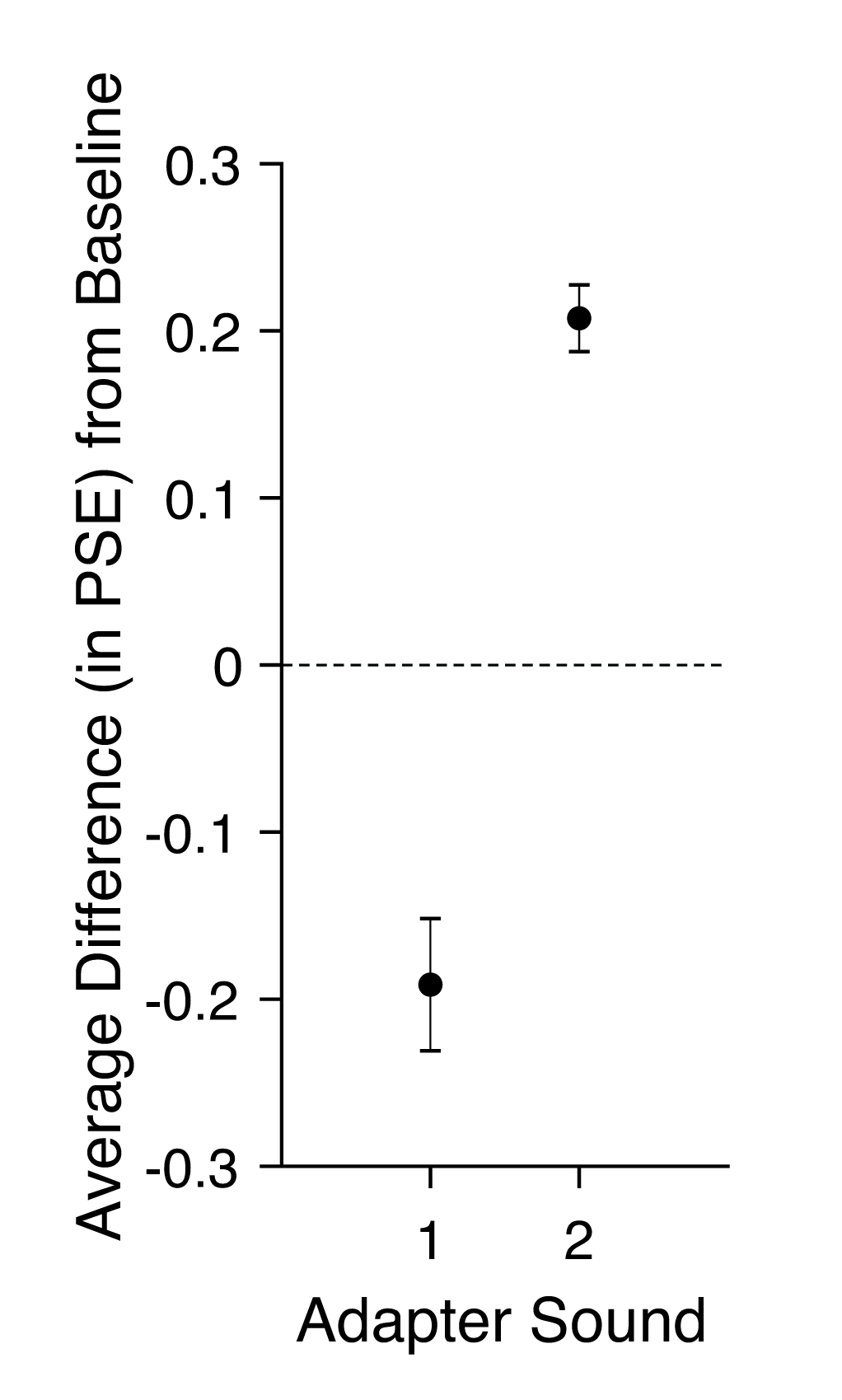


**Supplementary Figure S4**. To eliminate the possibility that the lack of adaptation to the “distilled centroid” adapters in Experiment 4b (see Figure 3D, right panel) was due to subjects’ failure to adapt to them at all, we confirmed that these artificial sounds were internally adaptable in Supplementary Experiment 1. Specifically, a separate group of participants were exposed to these “distilled centroid” sounds and demonstrated perceptual shifts when tested on morphs between the “distilled centroid” adapters themselves, rather than morphs between the original instrument sounds, as in Experiment 4b. N = 12. Error bars are SEM.

Legends for Supplementary Audio Files

Supplementary Audio S1. Clarinet (adapter sound, Experiment 1)

Supplementary Audio S2. Oboe (adapter sound, Experiment 1)

Supplementary Audio S3. Morph between a clarinet and an oboe (probe sound, Experiment 1)

Supplementary Audio S4. Female voice (adapter sound, Experiment 2)

Supplementary Audio S5. Male voice (adapter sound, Experiment 2)

Supplementary Audio S6. Morph between a male and female voice (probe sound, Experiment 2)

Supplementary Audio S7. Horse (adapter sound, Experiment 2)

Supplementary Audio S8. Crunching noise (adapter sound, Experiment 2)

Supplementary Audio S9. Morph between a horse and a crunching noise (probe sound, Experiment 2)

Supplementary Audio S10. Dog (adapter sound, Experiment 2)

Supplementary Audio S11. Goose (adapter sound, Experiment 2)

Supplementary Audio S12. Morph between a dog and a goose (probe sound, Experiment 2)

Supplementary Audio S13. Effect demonstration with instructions (Experiment 1)

Supplementary Audio S14. Example trial (Experiment 1, clarinet adapter)

Supplementary Audio S15. Example trial (Experiment 1, oboe adapter)

Supplementary Audio S16. Example trial (Experiment 3, oboe adapter, probe sound played on C5)

Supplementary References

53. Fitz, K., Haken, L., Lefvert, S., Champion, C., & O'Donnell, M. Cell-utes and flutter-tongued cats: Sound morphing using Loris and the reassigned bandwidth-enhanced model. *Comput. Music. J.* **27**, 44-65 (2003).

54. Polansky, L., & McKinney, M. Morphological mutation functions: Applications to motivic transformation and a new class of cross-synthesis techniques. *Proc. ICMC*, 234-241 (1991).

55. Polansky, L. More on morphological mutation functions: Recent techniques and

developments. *Proc. ICMC*, 57-60 (1992).

56. Kawahara, H., & Matsui, H. Auditory morphing based on an elastic perceptual distance metric in an interference-free time-frequency representation. *Proceedings of the IEEE International Conference on Acoustics, Speech, and Signal Processing (Hong Kong)*, 256–259 (2003).

57. Bruckert, L. et al. Vocal attractiveness increases by averaging. *Curr. Biol.* **20**, 116-120 (2010).

58. Wichmann, F. A., & Hill, N. J. The psychometric function: I. Fitting, sampling, and goodness of fit. *Percept. Psychophys*. **63**, 1293–1313 (2001).
